# Motives for Quitting Smoking and Reasons for Relapse Among Current Smoking Quitters in Abha City, Saudi Arabia: A Cross-Sectional Study

**DOI:** 10.3390/healthcare14142030

**Published:** 2026-07-08

**Authors:** Sultan Abdullah Albqami, Ali Hassan Almaqsudi, Hajar Saeed Alqahtani, Nawaf Ahmed Alqahtani Alsaqer, Afnan Mohammad Aseeri, Abdullah Mohammed Abdullah Al-Qahtani, Abdulmohsen Mohammed Abdullah Al-Qahtani

**Affiliations:** 1 Department of Preventive Medicine, Jazan Health Cluster, Jazan 45142, Saudi Arabia; 2 Tobacco Control Program, Aseer Health Cluster, Abha 62523, Saudi Arabia; alim@moh.gov.sa (A.H.A.); halqahtani75@moh.gov.sa (H.S.A.); 3 Department of Preventive Medicine, Aseer Central Hospital, Aseer Health Cluster, Abha 62523, Saudi Arabia; dr.nawaf48alsaqer@gmail.com (N.A.A.A.); afnan.asiri09@gmail.com (A.M.A.); 4 Department Preventive Medicine, Armed Forces Hospital Southern Region, Khamis Mushayt 62413, Saudi Arabia; amaq1030@gmail.com; 5 Department of Physical Therapy, General Department of Medical Services, Ministry of Interior, Riyadh 13321, Saudi Arabia; abq140555@gmail.com

**Keywords:** tobacco smoking, smoking cessation, nicotine dependence, relapse, Saudi Arabia

## Abstract

**Background:** Tobacco smoking cessation is a complex process influenced by biological, psychological, and social factors. Despite the availability of effective cessation interventions, relapse remains common among smokers attempting to quit. This study aimed to identify factors associated with previous smoking relapse and nicotine dependence among smokers attending anti-smoking clinics in Abha City, Saudi Arabia. **Methods:** An analytical comparative cross-sectional study was conducted among 330 adult Saudi smokers attending the Abha Anti-smoking Clinic and the Erada Anti-smoking Clinic. Previous smoking relapse was defined as having one or more unsuccessful smoking cessation attempts after a period of abstinence. Data were collected using a structured interviewer-administered questionnaire covering sociodemographic characteristics, smoking history, nicotine dependence, withdrawal symptoms, motives for smoking cessation, and relapse-related factors. Nicotine dependence was assessed using the Fagerström Test for Nicotine Dependence (FTND). Descriptive statistics, chi-square tests, and multivariable logistic regression analyses were performed. **Results:** Most participants were male (84.2%) and aged 20–39 years (59.4%). Smoking initiation most commonly occurred between 15 and 20 years of age (68.8%), while 66.4% reported previous quit attempts. Nervousness (84.2%) and stress or tension (74.2%) were the most frequently reported withdrawal symptoms. High and very high nicotine dependence were reported by 26.1% and 14.2% of participants, respectively. High nicotine dependence (AOR = 1.39, 95% CI: 1.15–1.69, *p* = 0.001) and very high nicotine dependence (AOR = 1.62, 95% CI: 1.21–2.17, *p* < 0.001) were independently associated with greater odds of previous smoking relapse. Attendance at the Erada Anti-smoking Clinic was associated with lower odds of relapse (AOR = 0.52, 95% CI: 0.29–0.91, *p* = 0.021). **Conclusions:** Higher nicotine dependence was independently associated with previous smoking relapse among smokers attending anti-smoking clinics. Comprehensive cessation programs integrating behavioral counseling, pharmacological treatment, and sustained follow-up may improve long-term abstinence and reduce relapse.

## 1. Introduction

Tobacco smoking remains one of the leading preventable causes of morbidity and mortality worldwide, contributing significantly to cancer, cardiovascular diseases, stroke, and chronic respiratory conditions [1,2,3]. Despite widespread awareness of its health risks, smoking continues to be highly prevalent, reflecting the complex interaction between biological addiction, behavioral patterns, and social influences [4]. Nicotine, the primary addictive component of tobacco, produces dependence through its psychoactive and reinforcing effects, making cessation difficult even among individuals with established smoking-related diseases [4]. Nicotine exerts its addictive effects primarily through activation of nicotinic acetylcholine receptors in the mesolimbic dopamine reward pathway. Repeated nicotine exposure stimulates dopamine release within the nucleus accumbens, reinforcing smoking behavior and contributing to the development of dependence. Over time, neuroadaptations occur that promote craving, withdrawal symptoms, and continued tobacco use despite awareness of its harmful health consequences [4].

Although many smokers express a willingness to quit, maintaining long-term abstinence remains challenging. Evidence suggests that approximately 58.6% of smokers attempt to quit for at least one day; however, nearly 80% relapse within the first month and up to 97% relapse within six months [5]. Long-term relapse rates can reach 77% to 92% within one year, indicating the chronic relapsing nature of nicotine dependence [6]. These findings highlight the need to better understand the determinants of both successful cessation and relapse.

Smoking cessation is a dynamic, multi-stage process that involves progression from pre-contemplation to maintenance. This process is frequently hindered by physiological dependence, withdrawal symptoms, psychological stress, and social and environmental influences [7]. Commonly reported barriers include cravings, irritability, sleep disturbances, and fear of weight gain, while external factors such as peer influence, exposure to smokers, limited access to cessation services, and inadequate professional support further complicate quitting efforts [8].

Several interventions have been developed to support smoking cessation, including pharmacological therapies such as nicotine replacement therapy, bupropion, and varenicline, in addition to behavioral counseling approaches [9]. Nicotine replacement therapy reduces withdrawal symptoms by providing controlled doses of nicotine without exposure to tobacco smoke, while bupropion acts by modulating dopaminergic and noradrenergic neurotransmission. Varenicline is a partial agonist of the α4β2 nicotinic acetylcholine receptor and reduces both nicotine craving and the rewarding effects of smoking. Combining pharmacological and behavioral interventions has been shown to improve cessation outcomes, although long-term abstinence rates remain suboptimal [10].

In Saudi Arabia, recent evidence highlights the continued public health burden of tobacco use. A large cross-sectional study conducted in Riyadh reported that the prevalence of active smoking was 17.3%, while passive smoking affected 16.5% of adults. Male gender, larger household size, and lower income were identified as important predictors of smoking behavior [11]. Trends among Saudi youth also demonstrate concerning patterns. Although overall tobacco use declined from 15.9% in 2007 to 9.4% in 2022, early smoking initiation increased, and intention to quit decreased among some groups, particularly females [12].

Smoking behavior within specific Saudi populations also remains notable. A study among a university community in Makkah reported current daily smoking among 11.6% of participants, with substantially higher prevalence among males and peer influence identified as a major factor for smoking initiation [13]. In addition, exposure to secondhand smoke continues to represent a public health concern, despite high awareness regarding its harmful effects [14].

Previous studies have identified health concerns, financial burden, family influence, and social pressure as important motivators for smoking cessation, whereas nicotine dependence, withdrawal symptoms, stress, and smoking-related social environments remain major barriers to sustained abstinence. However, evidence regarding predictors of smoking relapse among smokers attending cessation clinics in Saudi Arabia remains limited. A better understanding of these factors is essential for developing targeted and individualized interventions that improve long-term cessation outcomes and reduce relapse rates.

Therefore, this study aimed to assess motives for smoking cessation, identify factors associated with previous smoking relapse, and examine the relationship between nicotine dependence and participant characteristics among smokers attending anti-smoking clinics in Abha City, Saudi Arabia.

## 2. Materials & Methods

### 2.1. Study Design and Setting

This analytical comparative cross-sectional study was conducted in Abha City, the capital of the Aseer Region, Saudi Arabia. The study was carried out in the two main anti-smoking clinics in the city: the Abha Anti-smoking Clinic and the Anti-smoking Outpatient Clinic at Erada Mental Health Hospital. Both clinics provide free smoking cessation services, including behavioral counseling and pharmacological support, for Saudi and expatriate smokers seeking to quit smoking. According to official clinic records, the total number of Saudi attendants registered at both clinics during 2024 was approximately 2474 individuals. The inclusion of both centers allowed comparison between clinic populations and enhanced the representativeness of the study sample. The study aimed to identify factors associated with previous smoking relapse and nicotine dependence among adult smokers attending anti-smoking clinics in Abha City, Saudi Arabia.

### 2.2. Study Population

The study population included Saudi adult smokers attending the anti-smoking clinics during the study period. Inclusion criteria were Saudi smokers aged 18 years or older, attending either anti-smoking clinic during the study period, able to communicate in Arabic, and willing to provide informed consent. Only Saudi nationals were included to ensure a relatively homogeneous study population and because the study sought to evaluate relapse patterns within the Saudi population attending governmental smoking cessation services. Exclusion criteria included individuals younger than 18 years, non-Saudi attendees, and participants who were unable or unwilling to complete the interview questionnaire.

### 2.3. Sample Size Calculation

The sample size was estimated using the standard formula for cross-sectional studies, assuming a 95% confidence level, a 5% margin of error, and an expected prevalence of 50%, yielding a minimum required sample size of 384 participants. Because the study was conducted over a predefined two-month data collection period and included all eligible Saudi smokers attending the two participating anti-smoking clinics during that period, a total of 330 participants were recruited. Although this number was lower than the calculated sample size, it represented the maximum number of eligible participants who met the inclusion criteria and consented to participate during the study period. A convenience sampling approach was adopted, whereby all eligible Saudi smokers attending the two participating anti-smoking clinics during the study period were consecutively invited to participate until the end of the data collection period. Of the 330 participants, 225 (68.2%) were recruited from the Abha Anti-smoking Clinic during October 2025, and 105 (31.8%) were recruited from the Erada Anti-smoking Clinic during November 2025.

### 2.4. Data Collection Tool and Variables

Data were collected using a structured questionnaire developed after a thorough review of relevant literature. The questionnaire captured participants’ sociodemographic characteristics, including age, gender, marital status, educational level, employment status, monthly income, and body mass index. Information related to smoking behavior was also obtained, including age at smoking initiation, previous quit attempts, duration of abstinence before relapse, and exposure to smoking within the household. Nicotine dependence was assessed using the Fagerström Test for Nicotine Dependence (FTND), and participants were asked about withdrawal symptoms experienced during smoking cessation attempts. In addition, the questionnaire explored motives for smoking cessation, perceived causes of relapse, and previous smoking relapse history.

The primary outcome variable was previous smoking relapse, operationally defined as self-reported resumption of tobacco smoking after at least one previous quit attempt following a period of abstinence. Relapse status was determined based on participants’ smoking history obtained during the interview and was not verified using biochemical measures such as exhaled carbon monoxide or cotinine testing.

### 2.5. Assessment of Nicotine Dependence

Nicotine dependence was assessed using the Fagerström Test for Nicotine Dependence (FTND), a validated six-item instrument widely used to measure the severity of physical dependence on nicotine [15]. The FTND evaluates key aspects of smoking behavior, including time to first cigarette after waking, number of cigarettes smoked per day, and difficulty refraining from smoking in restricted areas [16,17].

Scores range from 0 to 10, with higher scores indicating greater dependence. Participants were categorized into five levels of nicotine dependence as follows: very low (0–2), low (3–4), moderate (5), high (6–7), and very high (8–10). The Arabic version of the Fagerström Test for Nicotine Dependence (FTND), which has demonstrated acceptable reliability with a Cronbach’s alpha of approximately 0.68 and evidence of cross-cultural validity among Arabic-speaking populations, was used in this study [18].

### 2.6. Validity and Pilot Testing

The questionnaire was reviewed by a panel of senior academic staff in Community Medicine to assess face and content validity. Necessary modifications were made based on their feedback to ensure clarity and comprehensiveness. A pilot study was conducted on 20 participants attending the clinic to evaluate the clarity and consistency of the questionnaire. Participants completed the questionnaire twice, one week apart, to assess test–retest reliability. Data from the pilot study were not included in the final analysis.

### 2.7. Data Collection Procedure

Data were collected during routine clinic working days through face-to-face interviews conducted by the researcher with the assistance of the clinic physician. After explaining the study objectives, informed consent was obtained from all participants prior to enrollment. Participants completed the questionnaire and underwent routine clinical assessment. They also received standard smoking cessation support, including counseling and pharmacotherapy as appropriate. Participants were interviewed during clinic attendance, and information regarding previous quit attempts, relapse history, and duration of abstinence before relapse was obtained through self-report.

To minimize social desirability bias, participants were assured that their responses would remain confidential and would not affect the healthcare services they received. Interviews were conducted in a private setting whenever possible.

### 2.8. Statistical Analysis

Data were entered, cleaned, and analyzed using the Statistical Package for Social Sciences (SPSS), version 29 (IBM Corp., Armonk, NY, USA). Data cleaning included checking for completeness, consistency, and outliers prior to analysis. Descriptive statistics were used to summarize participants’ characteristics and study variables. Categorical variables were presented as frequencies and percentages, while continuous variables were summarized using means and standard deviations or medians and interquartile ranges, as appropriate based on data distribution. Inferential analysis was conducted to examine associations between participant characteristics and nicotine dependence levels. The chi-square test (or Fisher’s exact test when expected cell counts were less than five) was used to assess associations between categorical variables. Effect sizes for chi-square analyses were assessed using Cramer’s V and interpreted according to Cohen’s criteria.

Multivariable logistic regression analysis was performed to identify factors independently associated with previous smoking relapse. Variables were selected for inclusion in the multivariable logistic regression model based on their clinical relevance and evidence from previous literature regarding their potential association with smoking relapse. Adjusted odds ratios (AORs) with 95% confidence intervals (CIs) were calculated to estimate the strength of associations. Model fit was assessed using the Omnibus test of model coefficients, the Hosmer–Lemeshow goodness-of-fit test, Nagelkerke R^2^, and overall classification accuracy. All statistical tests were two-tailed, and a *p*-value of less than 0.05 was considered statistically significant.

### 2.9. Ethical Considerations

Ethical approval for this study was obtained from the Institutional Review Board (IRB) of the Aseer Health Cluster (Approval No. F3-1-2025). The study was conducted in accordance with the ethical principles of the Declaration of Helsinki. Participation was entirely voluntary, and written informed consent was obtained from all participants before enrollment. Prior to participation, all individuals were informed about the objectives of the study, the voluntary nature of participation, their right to withdraw at any stage without affecting the healthcare services they received, and the confidentiality of the collected information. All questionnaires were anonymized, and no personally identifiable information was recorded. The collected data were stored securely, accessed only by the research team, and used exclusively for research purposes.

## 3. Results

### 3.1. Sociodemographic and Smoking-Related Characteristics

A total of 330 participants were included in the study (Table 1). Most participants were aged between 20 and 39 years (59.4%), while 25.1% were aged 40 years or older and 15.5% were younger than 20 years. The majority of participants were male (84.2%), and slightly more than half were single (52.7%). Most participants had a university-level education (71.5%), whereas only 3.3% had primary or intermediate education. More than half of the participants were employed (53.9%), followed by students (26.1%). Regarding monthly income, 51.5% reported earning less than 10,000 SAR per month.

Nearly half of the participants had normal body mass index (49.4%), while 30.9% were overweight and 14.8% were obese. Most participants attended the Abha Anti-smoking Clinic (68.2%). Smoking initiation commonly occurred between the ages of 15 and 20 years (68.8%), while 11.5% reported starting smoking before the age of 15 years. Approximately half of the participants (49.4%) lived with other smokers.

Regarding smoking cessation history, 66.4% of participants had at least one previous quit attempt. Among participants with previous quit attempts, 50.7% reported three or more attempts. However, sustained abstinence remained limited, as 38.4% reported abstinence for less than one month before relapse, whereas only 14.6% maintained abstinence for more than six months.

### 3.2. Motives for Smoking Cessation, Relapse, and Withdrawal Symptoms

The most commonly reported motives for smoking cessation were considering smoking an unhealthy habit (86.4%), financial concerns related to smoking (78.2%), and the desire to improve health (77.3%) (Table 2). Other frequently reported motives included avoiding the unpleasant odor of smoking (75.2%), concerns regarding reduced immunity (74.2%), impaired fertility (68.5%), and accelerated aging appearance (63.0%).

**Figure 1:**
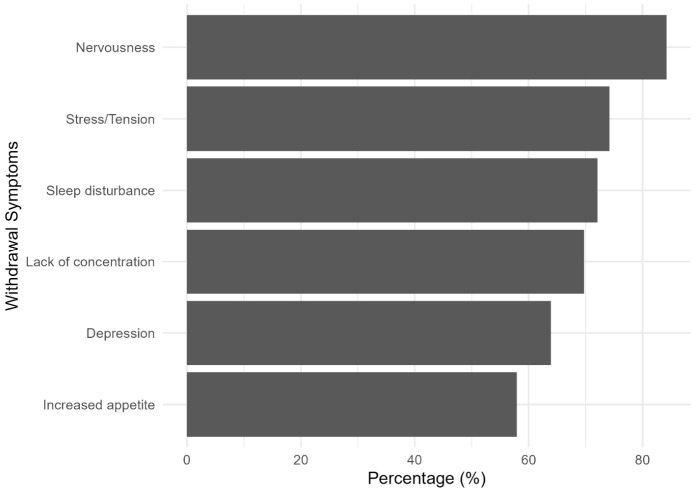
Prevalence of reported withdrawal symptoms among participants during smoking cessation attempts (*n* = 330).

Withdrawal symptoms represented the most commonly reported source of relapse (83.3%), followed by exposure to smoking friends or family members (69.4%), lack of motivation to quit smoking (61.8%), and lack of support from community members (57.3%). Increased body weight after smoking cessation was reported by 47.6% of participants as a contributing factor to relapse. Regarding withdrawal symptoms experienced during smoking cessation attempts, nervousness was the most frequently reported symptom (84.2%), followed by stress or tension (74.2%), sleep disturbances (72.1%), and lack of concentration (69.7%). Depression and increased appetite were reported by 63.9% and 57.9% of participants, respectively (Figure 1).

### 3.3. Distribution of Nicotine Dependence

**Figure 2:**
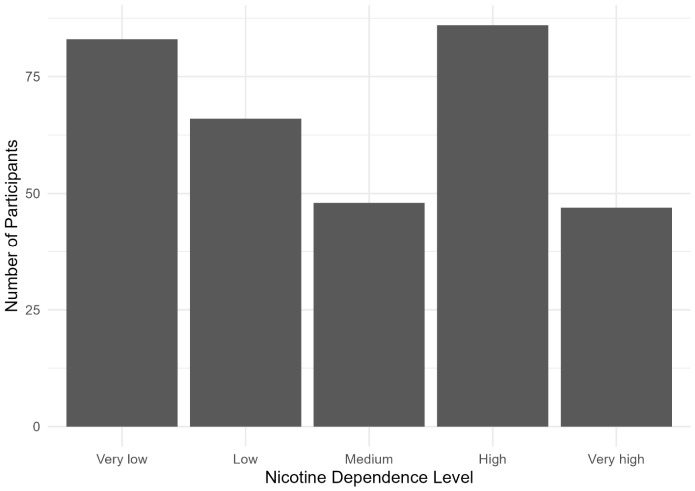
Distribution of nicotine dependence levels among study participants (*n* = 330).

Nicotine dependence levels varied among participants. High nicotine dependence was the most common category (26.1%), followed by very low dependence (25.2%) and low dependence (20.0%). Medium dependence was reported by 14.5% of participants, while 14.2% had very high nicotine dependence (Table 3 and Figure 2).

### 3.4. Association Between Nicotine Dependence and Participants’ Characteristics

Significant associations were observed between nicotine dependence levels and several participant characteristics (Table 4). Higher nicotine dependence levels were significantly associated with older age groups (*p* < 0.001). Participants aged 40 years or older demonstrated higher proportions of high and very high nicotine dependence compared with younger age groups. The strength of this association was moderate (Cramer’s V = 0.28). Gender was also significantly associated with nicotine dependence (*p* < 0.001). Males showed markedly higher proportions of high and very high dependence levels compared with females. This association demonstrated a moderate effect size and represented the strongest association observed among the studied variables (Cramer’s V = 0.35). Similarly, married participants demonstrated higher nicotine dependence compared with single participants (*p* < 0.001), with a moderate association strength (Cramer’s V = 0.31).

Employment status showed a significant association with nicotine dependence (*p* < 0.001), with retired and employed participants demonstrating higher levels of dependence than students. The magnitude of this association was moderate (Cramer’s V = 0.26). Monthly income was also significantly associated with nicotine dependence (*p* = 0.029), although the effect size was relatively small (Cramer’s V = 0.17). Additionally, participants attending the Erada Anti-smoking Clinic showed higher proportions of very high nicotine dependence compared with those attending the Abha Anti-smoking Clinic (*p* = 0.033), with a small effect size (Cramer’s V = 0.16). No statistically significant association was observed between nicotine dependence and either educational level (*p* = 0.074; Cramer’s V = 0.14) or body mass index (*p* = 0.344; Cramer’s V = 0.09), indicating weak relationships between these variables and nicotine dependence.

### 3.5. Factors Associated with Previous Smoking Relapse

Previous smoking relapse was defined as self-reported resumption of tobacco smoking after a previous quit attempt following a period of abstinence. Multivariable logistic regression analysis showed that nicotine dependence level and anti-smoking center were independently associated with previous smoking relapse (Table 5). Compared with participants with very low or low nicotine dependence, those with high nicotine dependence had significantly greater odds of reporting previous relapse (adjusted OR = 1.39, 95% CI: 1.15–1.69, *p* = 0.001). The association was even stronger among participants with very high nicotine dependence (adjusted OR = 1.62, 95% CI: 1.21–2.17, *p* < 0.001).

In contrast, participants attending the Erada Anti-smoking Clinic had significantly lower odds of previous relapse compared with those attending the Abha Anti-smoking Clinic (adjusted OR = 0.52, 95% CI: 0.29–0.91, *p* = 0.021). No statistically significant associations were observed between previous smoking relapse and age group, gender, marital status, employment status, or monthly income after adjustment for potential confounding factors (all *p* > 0.05).

## 4. Discussion

This study investigated factors associated with smoking relapse and nicotine dependence among smokers attending two anti-smoking clinics in Abha City, Saudi Arabia. The findings are consistent with the Biopsychosocial Model of tobacco dependence, which proposes that smoking behavior and relapse arise from the complex interaction of biological, psychological, and social factors rather than a single determinant. In the present study, nicotine dependence represented the biological component, withdrawal symptoms and motivation reflected psychological influences, while exposure to smokers and clinic-related factors represented important social determinants. Together, these findings suggest that smoking cessation is a multifactorial process requiring integrated interventions that simultaneously address nicotine dependence, psychological challenges, and the social environment. Higher nicotine dependence was independently associated with greater odds of previous smoking relapse, whereas attendance at the Erada Anti-smoking Clinic was associated with lower odds of relapse. In addition, nicotine dependence levels varied significantly according to several participant characteristics, particularly gender, marital status, age, and employment status.

The sociodemographic profile of participants showed a predominance of males and young adults, which aligns with previous studies conducted in Saudi Arabia that report markedly higher smoking prevalence among males compared to females [12,13]. This disparity has been attributed to cultural and social norms that discourage smoking among women [14]. Accordingly, the findings of the present study primarily reflect smoking cessation experiences among Saudi men, and caution should be exercised when extrapolating these findings to female smokers, whose psychological, social, and cultural barriers to smoking cessation may differ [11]. In addition, most participants initiated smoking during adolescence, consistent with evidence indicating that smoking behavior often begins at a young age and is strongly associated with long-term dependence and lower cessation success [19]. These findings reinforce the importance of early prevention strategies, particularly school-based interventions, to reduce smoking initiation among adolescents [20].

Most participants reported previous attempts to quit smoking; however, long-term abstinence remained limited, with only a small proportion maintaining abstinence for more than six months before relapse. This finding reflects the chronic relapsing nature of nicotine addiction and is consistent with previous literature demonstrating that repeated quit attempts are common before achieving sustained cessation [21]. The high prevalence of withdrawal symptoms observed in this study, including nervousness, stress, and sleep disturbances, further supports the biological basis of addiction [22]. These symptoms reflect neuroadaptations that occur following nicotine cessation, whereby reduced stimulation of nicotinic acetylcholine receptors results in decreased dopamine release within the mesolimbic reward pathway, leading to craving, irritability, anxiety, impaired concentration, and sleep disturbances [4]. These neurobiological changes are particularly pronounced during the first weeks after quitting and represent a critical period during which smokers are highly vulnerable to relapse.

Previous studies have demonstrated that craving and withdrawal symptoms are among the strongest predictors of smoking relapse, especially during the early phases of cessation [23]. Similar observations have been reported in studies from other countries, where withdrawal symptoms and nicotine craving were identified as major barriers to maintaining abstinence despite strong motivation to quit [23]. Comparable findings have been reported in studies conducted in Qatar, South Korea, and other low- and middle-income countries, where withdrawal symptoms, nicotine craving, and psychological distress consistently emerged as major determinants of unsuccessful quit attempts despite strong motivation to stop smoking [8,21,23]. These consistent observations across different healthcare systems suggest that withdrawal symptoms remain one of the most universal barriers to sustained tobacco abstinence. Therefore, smoking cessation programs should prioritize intensive support during the early post-cessation period through evidence-based pharmacotherapy, behavioral counseling, and regular follow-up visits. Early identification and proactive management of withdrawal symptoms may reduce relapse risk and improve long-term abstinence, particularly among smokers with high nicotine dependence.

The social environment was another important determinant identified in this study. Nearly half of the participants reported living with smokers, and exposure to smoking family members or friends represented one of the most commonly reported causes of relapse. Similar findings have been reported in previous studies showing that smokers surrounded by other smokers are less likely to successfully quit smoking [24]. Social modeling and normalization of smoking behavior within families and peer groups may contribute to both smoking initiation and relapse [25]. Consequently, strengthening family and community support systems and promoting smoke-free environments may play a significant role in improving cessation outcomes [26].

Nicotine dependence demonstrated a strong association with relapse risk. Participants with higher grades of nicotine dependence had significantly higher odds of previous smoking relapse. This finding is consistent with previous evidence linking higher Fagerström scores to stronger addiction severity and reduced cessation success [27]. Furthermore, moderate associations were observed between nicotine dependence and gender, marital status, and age, suggesting that these characteristics may have practical relevance in identifying smokers at greater risk of severe nicotine dependence [28]. Moreover, high nicotine dependence was more common among older participants, males, married individuals, and employed or retired participants. These findings may reflect the cumulative effect of prolonged smoking exposure and established smoking habits over time. Assessment of nicotine dependence should therefore be considered a fundamental component of smoking cessation services to allow individualized treatment planning. Smokers with high dependence levels may benefit from intensive counseling, nicotine replacement therapy, and close clinical follow-up. Recent evidence also supports the effectiveness of pharmacological interventions such as nicotine replacement therapy, bupropion, and varenicline in improving cessation outcomes among smokers with high levels of dependence and psychiatric comorbidities, highlighting the importance of integrating pharmacotherapy within smoking cessation programs [28].

Motivation to quit smoking represented another important finding in the current study. The most frequently reported motivations included awareness of the harmful health effects of smoking, financial burden, and the desire to improve overall health and quality of life. These findings are in agreement with previous studies reporting that health concerns and economic factors are among the strongest motivators for smoking cessation [26,27]. Increasing awareness regarding smoking-related health risks and financial consequences may therefore improve smokers’ readiness to quit and encourage cessation attempts.

Psychological factors also contributed substantially to relapse. Withdrawal symptoms, stress, and lack of motivation were commonly reported as barriers to successful cessation. Previous research has similarly identified stress and psychological dependence as major contributors to smoking relapse [29]. Furthermore, concerns regarding weight gain and sleep disturbances were frequently reported among participants, which is consistent with studies identifying these symptoms as common challenges following smoking cessation [30]. Recent work has emphasized the importance of weight-related smoking behaviors and concerns regarding post-cessation weight gain, as these factors may undermine quit attempts and contribute to relapse among some smokers [31]. Addressing these concerns through psychological support and behavioral counseling may improve smoking cessation outcomes and reduce relapse rates.

Interestingly, participants attending the Erada Anti-smoking Clinic had lower odds of reporting previous relapse compared with those attending the Abha Anti-smoking Clinic. However, given the cross-sectional design of the present study, this finding should be interpreted cautiously and should not be interpreted as evidence that one clinic is inherently more effective than the other. The observed difference may instead reflect variations in participant characteristics, nicotine dependence severity, treatment intensity, follow-up practices, healthcare provider expertise, patient adherence, or other unmeasured confounding factors that were not captured in the present analysis. Accordingly, the association should be regarded as hypothesis-generating rather than confirmatory. Future prospective multicenter studies should investigate clinic-level characteristics, including counseling protocols, pharmacotherapy utilization, follow-up strategies, and service organization, to identify best practices that could be standardized across smoking cessation clinics in Saudi Arabia.

Although several sociodemographic variables such as age, gender, marital status, employment status, and income were not independently associated with relapse in the adjusted analysis, these factors may still indirectly influence smoking behavior through interactions with psychological and environmental determinants [26,27,32]. Consequently, smoking cessation interventions should adopt a comprehensive and individualized approach that simultaneously addresses nicotine dependence, withdrawal symptoms, psychological support, and social influences. Based on the present findings, smokers with high FTND scores, repeated quit attempts, prominent withdrawal symptoms, or continued exposure to smokers should be prioritized for intensive relapse-prevention interventions, including individualized behavioral counseling, evidence-based pharmacotherapy, and structured follow-up during the early post-cessation period. Furthermore, the observed differences between the two participating clinics suggest that evaluating and standardizing best clinical practices across smoking cessation services may further improve treatment outcomes. At the public health level, strengthening family-based counseling, promoting smoke-free home environments, and implementing school- and community-based programs aimed at delaying smoking initiation may contribute to reducing nicotine dependence and relapse among smokers in Saudi Arabia.

## 5. Limitations

Several limitations should be considered when interpreting the findings of this study. First, the cross-sectional design limits the ability to establish temporal relationships or infer causality between the investigated factors and smoking relapse. Consequently, the observed associations should be interpreted as correlational rather than causal. Furthermore, the temporal sequence between nicotine dependence, withdrawal symptoms, psychosocial factors, and smoking relapse cannot be determined. For example, although higher nicotine dependence was associated with previous smoking relapse, it is not possible to establish whether greater nicotine dependence increased the likelihood of relapse or whether repeated unsuccessful quit attempts contributed to higher levels of dependence. Similarly, withdrawal symptoms and psychological distress may represent both antecedents and consequences of relapse. Therefore, the observed associations should be interpreted as hypothesis-generating and confirmed in future longitudinal studies.

Second, the study relied on self-reported information regarding smoking behavior, previous quit attempts, relapse history, and withdrawal symptoms, which may be subject to recall bias and social desirability bias. Recall bias may have particularly affected participants’ reporting of previous quit attempts, duration of abstinence, and reasons for relapse, especially among individuals who had experienced multiple cessation attempts over several years. Although participants were assured of confidentiality and interviews were conducted in a supportive clinical environment, the possibility of reporting bias cannot be excluded.

Third, participants were recruited from only two anti-smoking clinics in Abha City, which may limit the generalizability of the findings to smokers who do not seek professional cessation support or to populations in other regions of Saudi Arabia. Moreover, participants attending specialized smoking cessation clinics are likely to differ from smokers in the general community with respect to motivation to quit, treatment-seeking behavior, and nicotine dependence. Therefore, the findings should not be generalized to all smokers in Saudi Arabia without appropriate caution. In addition, the use of convenience sampling may have introduced selection bias, as individuals willing to participate may differ systematically from those who declined participation or who do not seek cessation services.

The predominance of male participants (84.2%) may also have limited the ability to fully explore gender-specific factors associated with smoking cessation and relapse. Because female smokers remain underrepresented in the present study, the findings primarily reflect smoking cessation experiences among Saudi men and may not fully capture the unique psychological, social, and cultural barriers encountered by women attempting to quit smoking.

Furthermore, smoking relapse was determined through self-report and was not verified using biochemical measures such as carbon monoxide monitoring or cotinine testing. Consequently, some degree of misclassification of smoking status cannot be excluded. In addition, some potentially relevant factors, including psychiatric comorbidities, smoking duration, treatment adherence, and detailed measures of social support, were not comprehensively assessed. Although multivariable logistic regression analysis was performed, residual confounding resulting from unmeasured variables cannot be completely excluded.

Despite these limitations, the study provides valuable insight into factors associated with nicotine dependence and smoking relapse among smokers attending specialized cessation clinics. Future prospective multicenter studies involving larger and more diverse populations are warranted to better characterize predictors of long-term smoking abstinence and relapse, validate these findings across different healthcare settings in Saudi Arabia, and evaluate causal pathways through longitudinal follow-up.

## 6. Conclusions

Smoking relapse remains a substantial challenge among smokers seeking cessation support and appears to be influenced by multiple biological, psychological, and social factors. The present study demonstrated that high and very high nicotine dependence were independently associated with increased odds of previous smoking relapse, highlighting the importance of systematically assessing nicotine dependence in smokers seeking cessation services. Withdrawal symptoms, particularly nervousness, stress, sleep disturbances, and impaired concentration, were highly prevalent and represented major perceived barriers to sustained abstinence. In contrast, health concerns and financial burden emerged as the strongest motivations for quitting tobacco smoking. These findings suggest that smoking cessation services should prioritize early identification of smokers with high FTND scores, provide tailored behavioral counseling and evidence-based pharmacotherapy according to the severity of nicotine dependence, and strengthen structured relapse-prevention follow-up during the first months after smoking cessation, when withdrawal symptoms are most prominent. Given the important influence of social exposure to smoking, family-centered counseling and interventions that promote smoke-free home environments should also be incorporated into routine cessation programs. From a public health perspective, policymakers should consider standardizing smoking cessation protocols across anti-smoking clinics, expanding access to cessation medications, strengthening relapse-prevention services, and implementing school- and community-based interventions aimed at preventing smoking initiation during adolescence. Future prospective multicenter studies are warranted to validate these findings and identify causal pathways underlying smoking relapse in Saudi Arabia.

## Figures and Tables

**Table 1:** Sociodemographic and smoking-related characteristics of participants (*n* = 330).

Characteristic	Category	*n*	%
Age group (years)	<20	51	15.5
	20–39	196	59.4
	≥40	83	25.1
Gender	Male	278	84.2
	Female	52	15.8
Marital status	Single	174	52.7
	Married	156	47.3
Educational level	Primary	4	1.2
	Intermediate	7	2.1
	Secondary	83	25.2
	University	236	71.5
Employment status	Unemployed	38	11.5
	Employed	178	53.9
	Student	86	26.1
	Retired	28	8.5
Monthly income (SAR)	<10,000	170	51.5
	10,000–20,000	112	33.9
	>20,000	48	14.5
Body mass index	Underweight	16	4.8
	Normal	163	49.4
	Overweight	102	30.9
	Obese	49	14.8
Anti-smoking center	Abha Anti-smoking Clinic	225	68.2
	Erada Anti-smoking Clinic	105	31.8
Age at first cigarette (years)	<15	38	11.5
	15–20	227	68.8
	>20	65	19.7
Living with smokers	Yes	163	49.4
Previous quit attempts	None	111	33.6
	One	47	21.5
	Two	61	27.9
	Three or more	111	50.7
Duration of last abstinence before relapse (*n* = 219)	<1 month	84	38.4
	1–2 months	72	32.9
	3–6 months	31	14.2
	>6 months	32	14.6

Note: Values are presented as number and percentage. Percentages were calculated based on the total sample size (*n* = 330), except for duration of last abstinence before relapse, which was calculated among participants with previous quit attempts (*n* = 219). SAR: Saudi Arabian Riyal.

**Table 2:** Participants’ reported motives for smoking cessation, sources of relapse, and withdrawal symptoms (*n* = 330).

Variable	Category	*n*	%
Motives for smoking cessation	Smoking is an unhealthy habit	285	86.4
	Smoking is a waste of money	258	78.2
	To improve health	255	77.3
	To avoid the bad odor of smoking	248	75.2
	Smoking lowers immunity	245	74.2
	Smoking impairs fertility	226	68.5
	Smoking makes me look older	208	63.0
Sources of relapse	Withdrawal symptoms	275	83.3
	Friends/family members who smoke nearby	229	69.4
	Lack of motivation to quit smoking	204	61.8
	Lack of support from community members	189	57.3
	Increased weight after quitting	157	47.6
Withdrawal symptoms experienced during cessation	Nervousness	278	84.2
	Tension or stress	245	74.2
	Sleep disturbances	238	72.1
	Lack of concentration	230	69.7
	Depression	211	63.9
	Increased appetite	191	57.9

Note: Values are presented as number and percentage. Participants were allowed to report more than one motive, source of relapse, and withdrawal symptom; therefore, percentages may exceed 100%.

**Table 3:** Distribution of nicotine dependence levels among participants (*n* = 330).

Nicotine Dependence Level	*n*	%
Very low	83	25.2
Low	66	20.0
Medium	48	14.5
High	86	26.1
Very high	47	14.2

Note: Nicotine dependence was assessed using the Fagerström Test for Nicotine Dependence (FTND). Values are presented as numbers and percentages.

**Table 4:** Association between nicotine dependence levels and participants’ characteristics (*n* = 330).

Characteristic	Very Low *n* (%)	Low *n* (%)	Medium *n* (%)	High *n* (%)	Very High *n* (%)	*p*-Value	Cramer’s V
Age group (years)						<0.001 ‡	0.28
<20	26 (51.0)	14 (27.5)	3 (5.9)	7 (13.7)	1 (2.0)		
20–39	44 (22.4)	40 (20.4)	26 (13.3)	58 (29.6)	28 (14.3)		
≥40	13 (15.7)	12 (14.5)	19 (22.9)	21 (25.3)	18 (21.7)		
Gender						<0.001 ‡	0.35
Male	55 (19.2)	59 (20.6)	47 (16.4)	80 (27.9)	46 (16.0)		
Female	28 (65.1)	7 (16.3)	1 (2.3)	6 (14.0)	1 (2.3)		
Marital status						<0.001 ‡	0.31
Single	63 (36.2)	36 (20.7)	12 (6.9)	47 (27.0)	16 (9.2)		
Married	20 (12.8)	30 (19.2)	36 (23.1)	39 (25.0)	31 (19.9)		
Educational level						0.074	0.14
Primary	0 (0.0)	0 (0.0)	1 (25.0)	0 (0.0)	3 (75.0)		
Intermediate	3 (42.9)	2 (28.6)	0 (0.0)	1 (14.3)	1 (14.3)		
Secondary	21 (25.3)	19 (22.9)	8 (9.6)	21 (25.3)	14 (16.9)		
University	59 (25.0)	45 (19.1)	39 (16.5)	64 (27.1)	29 (12.3)		
Employment status						<0.001 ‡	0.26
Unemployed	11 (28.9)	8 (21.1)	3 (7.9)	9 (23.7)	7 (18.4)		
Employed	29 (16.3)	33 (18.5)	36 (20.2)	52 (29.2)	28 (15.7)		
Student	42 (48.8)	21 (24.4)	5 (5.8)	15 (17.4)	3 (3.5)		
Retired	1 (3.6)	4 (14.3)	4 (14.3)	10 (35.7)	9 (32.1)		
Monthly income (SAR)						0.029 ‡	0.17
<10,000	54 (31.8)	34 (20.0)	19 (11.2)	38 (22.4)	25 (14.7)		
10,000–20,000	18 (16.1)	20 (17.9)	23 (20.5)	38 (33.9)	13 (11.6)		
>20,000	11 (22.9)	12 (25.0)	6 (12.5)	10 (20.8)	9 (18.8)		
Body mass index						0.344	0.09
Underweight	5 (31.3)	4 (25.0)	1 (6.3)	4 (25.0)	2 (12.5)		
Normal	48 (29.4)	33 (20.2)	24 (14.7)	41 (25.2)	17 (10.4)		
Overweight	23 (22.5)	15 (14.7)	18 (17.6)	27 (26.5)	19 (18.6)		
Obese	7 (14.3)	14 (28.6)	5 (10.2)	14 (28.6)	9 (18.4)		
Anti-smoking center						0.033 ‡	0.16
Abha Anti-smoking Clinic	53 (29.0)	42 (23.0)	24 (13.1)	46 (25.1)	18 (9.8)		
Erada Anti-smoking Clinic	30 (20.4)	24 (16.3)	24 (16.3)	40 (27.2)	29 (19.7)		

Note: Values are presented as numbers and percentages. *p*-values were calculated using the chi-square test. Effect sizes are reported using Cramer’s V, where values of approximately 0.10, 0.30, and 0.50 indicate small, moderate, and large effect sizes, respectively. Statistical significance was considered at *p* < 0.05. ‡ Statistically significant association. SAR: Saudi Arabian Riyal.

**Table 5:** Multivariable logistic regression analysis of factors associated with previous smoking relapse among participants (*n* = 330).

Variable	Category	Adjusted OR	95% CI	*p*-Value
Age group (years)	<20	1.00 (Ref)	–	–
	20–39	1.12	0.81–1.56	0.485
	≥40	1.25	0.93–1.68	0.147
Gender	Female	1.00 (Ref)	–	–
	Male	1.50	0.70–3.18	0.294
Marital status	Single	1.00 (Ref)	–	–
	Married	1.17	0.63–2.16	0.616
Employment status	Employed	1.00 (Ref)	–	–
	Unemployed	1.08	0.71–1.64	0.712
	Student	0.93	0.68–1.27	0.636
	Retired	1.15	0.75–1.76	0.518
Monthly income (SAR)	<10,000	1.00 (Ref)	–	–
	10,000–20,000	1.11	0.79–1.55	0.543
	>20,000	1.25	0.88–1.77	0.222
Grade of nicotine dependence	Very low/Low	1.00 (Ref)	–	–
	Medium	1.18	0.85–1.64	0.321
	High	1.39	1.15–1.69	0.001 ‡
	Very high	1.62	1.21–2.17	<0.001 ‡
Anti-smoking center	Abha Anti-smoking Clinic	1.00 (Ref)	–	–
	Erada Anti-smoking Clinic	0.52	0.29–0.91	0.021 ‡

Note: Adjusted odds ratios (ORs) and 95% confidence intervals (CIs) were obtained using multivariable logistic regression analysis. Reference categories (Ref) are explicitly defined for all categorical and ordinal variables. Statistical significance was considered at *p* < 0.05. ‡ Statistically significant association. Model fit statistics: Omnibus χ^2^(13) = 48.64, *p* < 0.001; Hosmer–Lemeshow χ^2^(8) = 7.42, *p* = 0.492; Nagelkerke R^2^ = 0.172; overall classification accuracy = 68.5%.

## Data Availability

The study data will be made available while removing any identifying or sensitive information to ensure participant confidentiality and compliance with ethical considerations.
